# Taking Practical Steps: A Feminist Participatory Approach to Cocreating a Trauma- and Violence-Informed Physical Activity Program for Women

**DOI:** 10.1177/10778012221134821

**Published:** 2022-11-10

**Authors:** Francine E. Darroch, Colleen Varcoe, Gabriela Gonzalez Montaner, Jessica Webb, Michelle Paquette

**Affiliations:** 1 Carleton University, Ottawa, ON, Canada; 2 University of British Columbia, Vancouver, BC, Canada; 3 Community Partner Organization, Vancouver, BC, Canada; 4 Community Advisory Board, Vancouver, BC, Canada

**Keywords:** trauma- and violence-informed care, trauma- and violence-informed physical activity, participatory action research, feminist research, physical activity programming, women's health

## Abstract

Trauma- and violence-informed physical activity (TVIPA) is a feasible approach to improve access/engagement in physical activity for pregnant/parenting women with experiences of trauma. Through feminist participatory action research, 56 semistructured interviews were completed to understand TVIPA. Four themes were identified: (1) “I have to be on edge”: Trauma and violence pervade women's lives, (2) “It should be mandatory that you feel safe”: Emotional safety is essential, (3) “The opportunity to step up and be decision-makers and leaders”: Choice, collaboration, and connection create safety, and (4) “It's a good start for healing,” strengths-based and capacity building foster individual and community growth.

## Background

Physical activity may be one of the most important and underutilized health promotion strategies for women with histories of trauma and/or ongoing experiences of gender-based violence (GBV), particularly for those who live in marginalizing circumstances (Ponic et al., 2011) including pregnant and parenting individuals (Darroch et al., 2022). Structural barriers, such as living in poverty, are also key contributors to increased exposure to trauma—including GBV, crime, and other forms of assault and violence (Klest, 2012)—as well as decreased physical activity (Darroch et al., 2022). Indeed, researchers have identified important relationships between physical activity and positive health outcomes for individuals who have experienced trauma (Björkman & Ekblom, 2021; Levine & Land, 2016; Rhodes et al., 2016; Rosenbaum et al., 2015). Engaging in regular physical activity can decrease depression, anxiety, sleep disturbances, and other health conditions associated with trauma and/or posttraumatic stress disorder (PTSD; Goldstein et al., 2018; Rosenbaum et al., 2015). In a recent systematic review and meta-analysis, Björkman and Ekblom (2021) concluded that physical activity can be an effective adjunctive PTSD treatment and noted greater amounts of physical activity may result in more beneficial outcomes. Further, Pebole et al. (2022) suggest that physical activity may be particularly beneficial for the mental health of survivors of sexual violence. Despite the widely accepted benefits of physical activity, inequitable access to and uptake of physical activity persists (Clark et al., 2014; Darroch et al., 2020), decreasing the potential benefits for those who live in marginalizing circumstances, particularly pregnant and parenting women.

Gendered experiences of trauma and subsequent interventions have been explored (Covington, 2000, 2008). Women are differentially affected by their experiences and subsequent responses to experiences. Although not specific to pregnant women, researchers have observed that females who are diagnosed with PTSD are both less likely to exercise and are less represented in health promotion research than their male counterparts (Pebole & Hall, 2019). There is a paucity of research on physical activity with pregnant and/or parenting individuals who have experienced trauma (Darroch et al., 2022). This is particularly important given that most pregnant women do not meet the current physical activity recommendations, and many remain inactive during and after pregnancy (da Silva et al., 2016; DiPietro et al., 2019). Researchers have found that physical activity during pregnancy can reduce the risk of postpartum depression and antenatal anxiety and depressive symptomology (DiPietro et al., 2019). While the benefits of physical activity during and postpregnancy have been well-documented in the general population, it is crucial to note that pregnancy is a time point of heightened risk of intimate partner violence (Gürkan et al., 2020; Schaefer et al., 2021) necessitating the need for additional supports and gender-specific interventions.

In response to identified inequities, researchers, advocates, and policymakers have called for physical activity programs to adopt the key principles of trauma- and violence-informed care (TVIC) including trauma awareness, safety and trustworthiness, opportunity for choice and collaboration, and a strengths-based and capacity building approach (Darroch et al., 2020; Ponic et al., 2011). The goal of TVIC is to prevent retraumatization and promote feelings of resilience, control, and safety in individuals by educating care providers and individuals who work within healthcare and social service organizations (Government of Canada, 2018). A TVIC perspective throughout healthcare or social systems can bring awareness to the impact of trauma and violence on the lives of individuals and reduce harm (Browne et al., 2015; Government of Canada, 2018). TVIC does not require disclosures of trauma, nor is it intended to treat trauma symptomology (Browne et al., 2012). Rather, those who provide TVIC recognize that traumatic experiences shape an individual's identity and contribute to the ways they experience interactions, structures, and systems (Wathen et al., 2021). Trauma- and violence-informed approaches to care were initially developed within services for individuals accessing healthcare and substance use services (Covington, 2008; Drabble et al., 2013); however, the tenets of TVIC have been extended to a wider range of sectors including physical activity (Darroch et al., 2020).

### Trauma- and Violence-Informed Physical Activity

Trauma- and violence-informed physical activity (TVIPA) “is an approach that considers and accounts for intersecting effects of systemic, structural, and interpersonal violence in the development, implementation, and delivery of physical activity programs” (Darroch et al., 2023). Those who provide conventional physical activity programs seldom account for social and structural inequities; therefore, a TVIPA approach calls for physical activity providers to consider individual, institutional, structural, and systemic issues. TVIPA has adopted the four key tenets from TVIC: trauma awareness, safety and trustworthiness, opportunity for choice, collaboration and connection, and a strengths-based and capacity-building approach (Wathen & Varcoe, 2019).

### Trauma- and Violence-Awareness

An understanding of the widespread prevalence of trauma and PTSD in society is of paramount importance; in the Canadian context, 76% of adults have reported an experience of trauma in their lifetime (Van Ameringen et al., 2008) and approximately 40% of women ages 15 years of age and older have experienced an incidence of physical or sexual violence (Cotter & Savage, 2019). The impact of trauma and/or PTSD on individuals varies immensely as do potential coping strategies and behaviors, including engagement in physical activity. Encompassed within trauma- and violence-awareness is the awareness of structural violence. Rylko-Bauer and Farmer (2016) defined structural violence as “violence of injustice and inequity” (p. 47). This is deeply rooted in a systemic exclusion that disadvantages specific populations (Farmer et al., 2013). Attwood et al. (2016) argued that those with “less access to power, wealth or prestige may be less likely to engage in sufficient physical activity to benefit their health” (p. 2). Thus, a TVIPA approach requires all individuals in organizations and across institutional levels to develop an awareness of how racism, poverty, sexism, and other forms of disadvantage exclude some people from physical activity. There are obvious mechanisms of exclusion such as a lack of availability of safe and affordable activities, spaces, equipment, and guidance, as well as barriers such as intersecting forms of stigma related to poverty, mental illness, and substance use.

### Safety and Trustworthiness

A trauma- and violence-informed approach must emphasize safety, including emotional and cultural safety (Browne et al., 2012). Cultural safety is defined as care that is free from discrimination and offered in a manner that is respectful of a person's culture and beliefs; as such, it shifts responsibility to the provider and is used to attend to power imbalances (Browne et al., 2012). Individuals who have experienced violence and trauma often feel unsafe with others and unsafe in their bodies (Covington, 2008). Thus, physical activity delivered with a dominant approach (e.g., direct commands and physical contact without warning) has the potential to retraumatize those who have experienced trauma and/or violence (Weissbecker & Clark, 2007). The principles of TVIPA emphasize sensitivity to the vulnerabilities of trauma survivors and require that the delivery of programming be adapted in a way that avoids retraumatization (Ammann & Matuska, 2014). Finding safe, appropriate, appealing, and effective methods to engage all individuals is necessary to ensure equitable access to the benefits of physical activity.

### Opportunity for Choice, Collaboration, and Connection

Opportunities for choice and collaboration are essential aspects of TVIPA to ensure participants’ self-efficacy and control (Darroch et al., 2023). In addition to individual choice about program offerings, participants should have opportunities for collaboration as a strategy to address and mitigate power differentials (Browne et al., 2012). Participants must be recognized as experts on their own lives, bodies, as well as their choices and personal capacity. Concrete strategies to enhance opportunities for choice and collaboration include providing clear information and expectations, actively listening to participants, using supportive language, engaging with a nonjudgmental attitude, providing alternative techniques, and supporting participants’ own decision-making and the fact they may not want feedback (Darroch et al., 2023). Through physical activity participation, individuals may develop better coping skills and physiological resilience in a “safe” environment (Ammann & Matuska, 2014). Because those providing programs may also have histories of trauma and may experience vicarious trauma, these considerations must be extended to staff.

### Strengths-Based and Capacity Building

A trauma- and violence-informed approach is strengths-based and aims to build capacity for individuals, organizations, and communities. Ammann and Matuska (2014) suggest that trauma-informed approaches to physical activity have the potential to build social bonds and empower participants’ self-care and confidence, which in turn can strengthen individuals’ and communities’ abilities to cope with past, current, or future traumatic experiences. Leveraging physical activity as a strategy to address dysregulated stress responses (and co-regulatory responses, in the case of group activities) may be an effective strategy to build individual emotional and social capacity (Ammann & Matuska, 2014). In addition to physiological benefits, physical activity has also been identified as a strategy for improving social and community cohesion (Yip et al., 2016).

Existing trauma-informed approaches to physical activity programs point to positive physical and psychosocial outcomes for women (Emerson, 2015; Jindani & Khalsa, 2015; Rhodes, 2016; van der Kolk et al., 2014; West et al., 2017). There is a strong representation in the literature examining the implementation of trauma-sensitive yoga practices (Clark et al., 2014; Emerson, 2015; Jindani & Khalsa, 2015; Rhodes, 2016; Smoyer, 2016; van der Kolk et al., 2014; West et al., 2017). However, there is limited empirical research on TVIPA outside of yoga-based programming (Darroch et al., 2020). Further, to our knowledge, there is a paucity of research examining TVIPA among pregnant and parenting women who experience marginalization.

The current pilot study provided an opportunity to use the TVIC tenets in the cocreation of physical activity programming for women, evaluate the usefulness of the approach from the perspective of participants, and deepen understanding of the tenets. Our study aimed to address these gaps in the literature and answer the question, “how do pregnant and parenting women who live in marginalizing conditions with experiences of trauma and/or violence perceive and experience TVIPA across a range of physical activities?”

## Methodology/Theory/Methods

The presented work is part of a larger feminist participatory action research (FPAR) project to address equity issues related to physical activity for pregnant and parenting women^1^ in Vancouver, Canada's Downtown Eastside (DTES). We use the terms woman/women as inclusive of anyone who identifies as a woman. The DTES has been identified as the lowest-income neighborhood in Canada, and it is inhabited by approximately 18,000 residents (Campbell et al., 2009). Residents experience high rates of precarious housing, mental illness, communicable disease, violence, trauma, and substance use when compared to Vancouver's general population (Linden et al., 2013). Thirty-eight percent of the residents live within family structures, and single parents lead 24% of these families (City of Vancouver, 2013). For pregnant and parenting women living in the DTES, experiences of trauma and/or violence can contribute to multiple barriers to physical activity engagement (Darroch et al., 2022). As such, the development of TVIPA programming has been identified as a way to cultivate appropriate, accessible, and desirable physical activity resources (Darroch et al., 2023).

FPAR requires a commitment to authentically engage community members and promote positive social change (Gatenby & Humphries, 2000); therefore, a key focus of this work was to center the lived experiences and knowledge of women, commit to genuine collaboration in every aspect of the research process, coproduce knowledge, and directly support the women in physical activity by addressing individual and structural barriers. In other words, we aimed to democratize our research process (Jaggar, 2008). Every aspect of this work was guided by a community advisory board (CAB) comprised of six pregnant and/or parenting women with lived experience of trauma, two service providers, and one researcher. The CAB consulted with the broader community and provided insights from their peers during each meeting. The CAB suggested semistructured interviews in order to facilitate the varied and challenging schedules of individual participants. All research tools, including the semistructured interview guides, were cocreated with the CAB who sought insights from the broader community. Further, the interviews were conducted by a researcher, a CAB member, and a community partner; participants had the opportunity to select their interviewer preference. Interviews occurred with women at regular intervals and their feedback both from these interviews and from informal conversations during their participation was incorporated into the programming in an ongoing manner. As such, the iterative process, and opportunities for participants to reassess if their needs are being met with subsequent programming align with the tenets of FPAR (Singh et al., 2013). At each relevant stage of the research, findings were also shared in public forums that were promoted with partner organizations and among participants.

Reid and Gillberg (2014) note the importance of the action component of FPAR which indicates a catalyst for positive change created by using the findings of studies and work toward social justice. Participants played active roles in selecting physical activity programs and in the production of knowledge and dissemination (all presentations were coled with a community partner, participant, and researcher). Although beyond the scope of this specific paper, the participants were coproducers of research throughout each phase of this work including the dissemination and use of results as such, a collaborative photovoice project was created as well as a documentary on the program—all ideas that are driven by and responsive to community members. In fact, participants created an entirely new program and line of research focused on the needs of families, including fathers (see Darroch et al., 2023). Reid (2004) argued that FPAR is both a methodological and conceptual framework to study women's health research as part of the social justice agenda, as such we opted to use this in conjunction with an intersectional theoretical framework.

In line with FPAR, we utilized an intersectional theoretical framework to understand intersecting vectors of oppression and mitigate barriers to physical activity through codeveloped TVIPA programming. This theoretical framework has been recognized as an important model for health researchers as it allows for a thorough examination of the complex and interacting factors that shape health outcomes (Bowleg, 2012). Furthermore, an intersectional framework foregrounds how historical structural systems of patriarchy, White supremacy, colonialism, ableism, and heteronormativity are integrally related depending on historical and social context (Russo, 2019).

### Overview of Taking Steps Program

**Figure 1.**
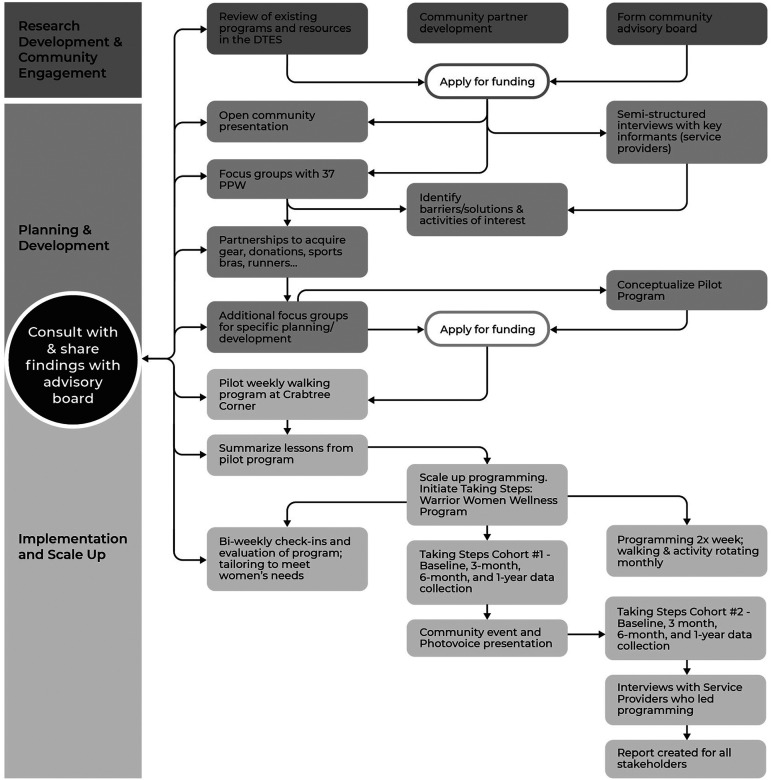
Overall Research Processes.

*Taking Steps,* a pilot program to develop and test TVIPA was initiated in May 2016 in accordance with FPAR principles. Over the course of four years, we developed partnerships, conducted environmental scans on existing physical activity programs/resources, completed semistructured interviews with service providers in the DTES, and ran focus groups to understand women's experiences, barriers, and enablers of physical activity. We then presented our findings to community members and various community organizations and ran additional focus groups to codevelop a community-based intervention (see Figure 1). To cocreate and refine a TVIPA pilot program, we purposefully worked to ensure community members were leaders, participants, advisors, and informants on adverse experiences and barriers to physical activity.

Given the socioeconomic status of most participants, addressing basic barriers to physical activity (e.g., appropriate workout clothing, equipment, and considerations for preworkout snacks) was of utmost importance. By securing funding and partnerships with various local organizations and companies, we were able to provide each participant with a pair of running shoes, socks, tank tops, hats, and water bottles. To address issues of food insecurity, we provided healthy snacks, water, and coffee before each activity to ensure participants had enough energy to exercise.

Physical activity programming was offered two times per week. The core activity was a weekly walking group that met once per week at 10:00 a.m., led by two staff members and the project coordinator; the starting location was our partner organization site (which offers support for pregnant/parenting women with substance abuse and/or those who have experienced abuse), known and trusted by most of the participating women. In addition to these weekly walks, we offered weekly physical activity programming that rotated monthly to address the needs and interests of participants that met once a week. Participants identified the activities that would be offered (e.g., noncontact boxing, kayaking, hiking, pow-wow dance, etc.), and we then sought instructors, provided TVIPA training, and engaged in an iterative process to provide and receive feedback on program delivery (see Figure 1). The programming ran consistently from May 2016 through March 2020, prior to the COVID-19 pandemic and program delivery is currently being reviewed and revised to meet local public health guidelines.

### Study Recruitment and Participants

Through the use of recruitment posters and snowball sampling, women who were pregnant and/or parenting and living or accessing services in the DTES were invited to participate in a physical activity program as well as the optional research study. As per participatory principles, women were welcome to take part in the activity program without any involvement in the research. As a result, over 100 women attended the programming and among these, 56 women participated in the research component. Participation in the walking program and additional activities ranged from 4 to 40 participants each week. Data were collected only from those consenting to participate in the research.

Participant eligibility included the following: 18 years or over (16 if legally emancipated); pregnant or within five years postpartum; live or access services in the DTES; able to understand English. All participants provided written informed consent prior to taking part in the research and received a $25 honorarium for each research assessment (including a survey and semistructured interview), on-site childcare, and bus tickets to attend research assessments. The 56 women ranged in age from 20 to 51; 59% (*n* = 33) identified as Indigenous. The women had given birth to a mean of 3.14 children and currently provided direct care to 1.82 children, demonstrating the overrepresentation of child apprehensions by the state. We purposely did not collect violence histories from the women to avoid the possibility of retraumatizing. Throughout the duration of the study, we learned all of the women had histories of trauma. All of the participants were accessing services from organizations that support individuals with histories of trauma and/or ongoing trauma, and substance abuse, and/or are precariously housed.

We drew on data from semistructured interviews with 56 pregnant and parenting women who took part in one of two cohorts of *Taking Steps: Warrior Women's Wellness* (Taking Steps) programming (see Table 1). The semistructured interviews were conducted across four assessments that took place at three-month intervals over a period of one year. The interviews included questions such as, “What has been the most enjoyable/effective part of the program? Can you discuss how this program supports women who have experienced violence and/or trauma? Since taking part in the program, how do you feel about accessing physical activity resources? What can the program do to better support women in the DTES?” The interviews lasted 10 to 60 min, were audio recorded, transcribed verbatim, and accuracy checked.

**Table 1.** Taking Steps Participant Demographics.

	Cohort 1 (*n*=34)	Cohort 2 (*n*=22)	Combined (*n*=56)
Age	34 (mean); 20–51 (range)	35 (mean); 28–46 (range)	35 (mean); 20–51 (range)
Identify as Indigenous	79.4% (*n* = 27)	30% (*n* = 6)	58.9% (*n* = 33)
Residential school survivor (for participants who identified as Indigenous) (*n*=33)			
No	25.9% (*n* = 7)	16.7% (*n* = 1)	24.2% (*n* = 8)
Attended	3.7% (*n* = 1)	—	3.0% (*n* = 1)
Parents attended	44.4% (*n* = 12)	50.0% (*n* = 3)	45.5% (*n* = 15)
Grandparents attended	22.2% (*n* = 6)	33.3% (*n* = 2)	24.2% (*n* = 8)
Data missing	3.7% (*n* = 1)	—	3.0% (*n* = 1)
Sexual orientation			
Heterosexual	85.3% (*n* = 29)	90.5% (*n* = 19)	87.7% (*n* = 48)
Bisexual	14.7% (*n* = 5)	4.8% (*n* = 1)	10.7% (*n* = 6)
Other	—	4.8% (*n* = 1)	1.8% (*n* = 1)
Currently in a relationship with partner	38.2% (*n* = 13)	61.9% (*n*=13)	46.4% (*n* = 26)
Highest level of education			
Elementary	2.9% (*n* = 1)	4.5% (*n* = 1)	3.6% (*n* = 2)
Some high school	52.9% (*n* = 18)	31.8% (*n* = 7)	44.6% (*n* = 25)
Completed high school	17.6% (*n* = 6)	9.1% (*n* = 2)	14.3% (*n* = 8)
Some college	26.5% (*n* = 9)	31.8% (*n* = 7)	28.6% (*n* = 16)
Completed college	—	18.2% (*n* = 4)	7.1% (*n* = 4)
Completed graduate or professional degree	—	4.5% (*n* = 1)	1.8% (*n* = 1)
Number of children given birth to	3.53 (mean); 1–11 (range)	2.55 (mean); 1–6 (range)	3.14 (mean); 1–11 (range)
Housing status			
Private apartment/condo/house	7.6% (*n* = 6)	31.8% (*n* = 7)	23.2% (*n* = 13)
Public, social, supportive housing	67.6% (*n* = 23)	59.1% (*n* = 13)	64.3% (*n* = 36)
Shelter	2.9% (*n* = 1)	—	1.8% (*n* = 1)
Other	11.8% (*n* = 4)	9.1% (*n* = 2)	10.7% (*n* = 6)

### Analysis

We employed Braun and Clarke's (2006) six-step approach to thematic data analysis. Participants were presented with the opportunity to take part in the analysis (and be compensated accordingly), however, only one individual opted to take part. Recognizing the constraints of the participants, namely time and capacity, participants thought it was reasonable to have one representative take part and share findings throughout the process. First, the authors and the representative from our CAB became familiar with the data by listening to recordings and reading and rereading transcripts. Second, authors one and three generated preliminary codes to provide further context to the themes. Third, we broke down the preliminary codes into overarching themes. Fourth, all of the authors met to discuss and reach a consensus on the themes and subthemes. Fifth, we defined and named themes, ultimately deciding to focus the findings on the tents of TVIPA with specific applications to physical activity. We presented our interpretation of the findings at a public session and asked for feedback from participants to see if the findings reflected what they experienced/saw in the community to ensure coproduction of knowledge. As our analysis shows, the themes aligned closely with the tenets of TVIPA and we illustrate that the tenets resonated strongly with the needs and perspectives of these participants. We argue that the tenets of TVIC promote health and well-being by increasing access to physical activity resources and programming for pregnant and parenting women.

## Findings

### “I Have to be on Edge”: Trauma and Violence Pervade Women's Lives

All of the participants described experiences of trauma and, for many, ongoing violence in their lives. The pervasiveness of trauma and violence in the women's lives and the ensuing impacts on their well-being pointed to the need for integrating trauma awareness into every level of programming including design and delivery. Participants described themselves as hypervigilant or “being on edge” in their daily lives and disclosed how these feelings carried over into any engagement with physical activity. In addition, over 90% (*n* = 51) of the participants reported experiencing discrimination (sometimes or often) in the past three months, reiterating the need for trauma awareness.

Participants noted that anyone involved in programming must be aware of the diverse impacts trauma can have on individuals in order to prevent harm. A woman highlighted the complexity of trauma and its invisibility: “you’re not going to know that there's going to be an issue until it comes up for a lot of people, I think that's the thing with trauma is lots of times you don't realize until you're there” (P8, 6-month assessment). Another woman followed up stating,

I’ve noticed that for a lot of us, there's triggers that you wouldn’t think about until you’re in the situation, so I think that if this program was to be replicated in other places, that is something that is pretty important to make the people who are running the program, facilitating them, to really be aware of … What might not bother one participant is going to be completely enough to ruin that activity for a person. (P16, baseline assessment)

Most women provided examples of trauma “triggers” that would “retraumatize” or “bother” themselves and others, including being unexpectedly touched, receiving commands, or experiencing abrupt changes to programming.

The women's key message was that anyone working with the community, including staff and volunteers, had to be aware of the complexity of trauma and ongoing violence and the varied ways in which these can impact women. For example, one participant commented on instructors at other fitness centers “and the ways that they motivate the group [yelling and telling people to push through pain], it can sometimes feel like you’re not doing a good enough job” (P8, 6-month assessment).

Participants specified that physical activity programmers should assume they will be dealing with the impacts of trauma. A participant shared,

All, every abuse, almost every abuse that is out there that is diagnosable, I had done to me: physical, emotional, sexual, yeah mentally, all those abuses … I find physical activity very challenging to get to because in my frame of mind all the time I have to be on edge. (P35, 3-month assessment)

During a discussion about barriers to physical activity, a participant explained the role that trauma and fear can play for women returning to physical activity and the importance of provider awareness: “there is fear of participating in … regular everyday activities that may have been removed from their regular routine to the point where they are too scared to go to the community centre to try and access a program” (P7, 3-month assessment).

For many women, fear emanated from and was compounded by the precarious nature of their lives, which included structural inequities including poverty, lack of housing, criminal justice or child protection, and immigration policies. A participant explained,

I have been jumping around from shelter to shelter … I ended up having PTSD. With such an overwhelming situation thinking I am going to have to fly back to [country of origin] and leave my son here, he's only a baby. That fear can drive you absolutely nuts and you almost just get into this tunnel vision of like, I need to figure out food, shelter, how to look after him before my physical or mental health. (P50, 3-month assessment)

She explained that she was not able to obtain a leisure access pass for free (or discounted) entry to recreational facilities because proof of residency was required, excluding individuals who are not permanent residents of Canada or those who do not have a fixed address. To ensure equal access, participants highlighted that it is imperative to consider their intersecting and diverse identities as mothers and women who have experienced trauma in the development and delivery of physical activity programs.

### “It Should be Mandatory That You Feel Safe”: Emotional Safety is Essential

The women emphasized the importance of safety and trustworthiness in the context of physical activity programming. They advised that ensuring safety should include enacting strategies to support women who may be triggered, while also engaging in programming with the intention to prevent such triggering. A participant shared,

When someone has some sort of traumatic issues come up, you can’t just walk away from it. You can’t be like “oh you’re fine, see you later, have a nice day.” You can’t leave a person in crisis like that. You can’t. So, anyone coming in to train us or to meet us for the first time, before you start working on a plan to get healthy in the gym or whatever it be, they need to know that; they can’t just come in, okay. (P6, baseline assessment)

Another participant suggested new physical activity leaders meet the women prior to the activity in their own space, as a strategy to increase safety and build trust. She stated,

They could come to the [Taking Steps] weekly walking group one time and then it would be a lot easier, you know, they could … introduce them, and then say here is the information … Then we would be more familiar when you go there. (P22, 3-month assessment)

For some participants, creating safety and trustworthiness also meant feeling supported in a nonjudgmental manner as they navigated new physical spaces. One participant detailed the discrimination she felt the last time she had attended a gym, explaining why she could not return:

My track marks, my scars—they are there, they show. People look and some people ask, and I am like f*ck, I can’t go back [to that gym]. Discrimination is everywhere. Especially for people who don’t know what it's like to be in recovery. It's huge and it sucks. (P21, baseline assessment)

Most of the women (93%) detailed experiences of discrimination in the prior three months that created barriers to accessing services: low education, income, substance use, mental health, and race were the most commonly cited reasons for discrimination. Approximately 59% of the study sample identified as Indigenous, 73% of whom reported being residential school^1^ survivors (either themselves, a parent, or a grandparent had attended residential school; see Table 1). Although data on citizenship status was not collected, a number of participants discussed the unique challenges of being a newcomer to Canada (immigrant or refugee status) and navigating the unique culture of the DTES. When discussing the creation of safe and trustworthy spaces, a participant suggested,

Ask participants if they need support navigating the centre or using equipment, maybe have more specific times when people could come in and say somebody is going to be here … it's a little bit still on the person to be like I don’t know how to use this stuff. (P12, baseline assessment)

The same participant also pointed out that you “need to meet people where they are at.” Many of the women had never attended structured physical activity programming, and they found it helpful and less stressful when clear information was provided about the walks or programs. Another participant reiterated the need for staff and volunteers to avoid judgment: “I think it's just really being a nonjudgmental person … like just really be there to support them.” One woman who attended the program further explained:

Being able to just have an all-women walking group and feel free and confident and non-judgmental about perhaps maybe the way we look or the way we dress, or things that are going on for us. I think that has been just getting to know the women has helped me meet other women just like me. (P12, 3-month assessment)

Because all the women had histories of trauma, many disclosed that they did not feel safe in mixed-gender spaces. The vast majority preferred “women's only” locations or times for being active. Throughout their interviews, most women described a safe environment as a key feature of the program.

### “The Opportunity to Step up and be Decision-Makers and Leaders”: Choice, Collaboration, and Connection may Contribute to Safety and Success of Programming

Participants emphasized that existing programs and resources did not meet their needs, and they therefore wanted to influence programming. Participants wanted to have their voices reflected in program development. A participant explained,

I think that there's a lot of different demographics that would benefit from this type [TVIPA] of programming, not just women or mothers … but I think that each organization would probably know what kind of activities are best, and especially in the Downtown Eastside, it really has to be influenced by the participants. (P8, 6-month assessment)

Reflecting on her experience attending the noncontact boxing program facilitated by Taking Steps, another woman emphasized the benefit of a participant-centered approach:

I don’t really like going to the gym, because it's too formal, kind of. I think the fun of [Taking Steps] is, it's kind of not super structured. Like, even at the boxing they weren’t like, “You have to do it.” Like, just try your best, you know. At the gym, everyone's staring at you, so it makes it more hard to do it. (P15, 6-month assessment)

As an advisory board member explained, “we have loved the opportunity to step up and be decision-makers and leaders in the program. We know all of the different challenges of raising children in the DTES” (P8, 12-month assessment).

The women had numerous practical suggestions. For example, although programs may be subsidized and seem affordable, they were out of reach for many women, leading them to argue for free, rather than subsidized programs. As one woman explained, “sometimes even $1.60 some days … that could be a lot—that is my kids’ snack at school” (P10, 3-month assessment). Another recommendation was to create more flexible programming so women could show up at any time during the designated session. The same participant explained that what works about Taking Steps is “[staff] having no judgements … I can show up 15 min late to [the activity] and not worry about like everyone going like hey [name] what's going on?”

Although the women represented a diversity of cultures, educational attainment, sexuality, and housing status, the shared experiences of parenting within the DTES context provided unique opportunities for women to connect. As another woman shared about her experience in the program, “it makes me more empathetic let's say, and also helps me to understand that I don’t have to be so hard on myself. So other than the exercise it's been more like the connection with the people” (P39, 3-month assessment). Social connections were consistently mentioned as a key outcome of the program. Another woman touched on this as well: “my confidence has gone up, I see women in the community that probably didn’t connect before, connect now because of the group … I have the ability to try new things without as much anxiety” (P10, 6-month assessment).

### “It's a Good Start for Healing”: Strengths-Based and Capacity Building Foster Individual and Community Growth

Participants repeatedly contrasted their prior experiences of physical activity programming with Taking Steps because of the nonjudgmental, strengths-based orientation. A woman explained,

So many other programs are about what we as parents are doing wrong and can be a reminder of the struggles we’ve gone through and the mistakes that we made but are already aware of. This group [Taking Steps] says, “come as you are and get in the moment.” It allows you to imagine a new and healthier way of living your life. (P10, 12-month assessment)

The participants wanted to create a system in which they felt supported to build on their strengths, including facilitating connections for other women to access new programs, resources, and locations. These new connections not only build on women's individual capacities, but also foster capacity building within their family and the greater community. As one woman explained,

I think having a group like this [Taking Steps] is really beneficial for women or other people who are maybe disadvantaged in any way, shape, or form, to build up the confidence to start accessing wellness, however that may look for them individually, but having this as a stepping-stone to get the courage to try and to apply for the leisure pass or whatever, take the kids to different programs and stuff. (P8, 3-month assessment)

Participants stressed the need to focus on women's strengths while acknowledging their experiences of trauma—without making it the focal point.

I think it's important to educate [program facilitators] about providing trauma-informed service, but not to focus on the trauma, but to focus on the positives that the women have accomplished. I think that's the really important thing, especially for someone who if they have no experience dealing with people with trauma. It's going to give them a biased idea if they’re only focusing on the negative side, the trauma, the violence, or what's caused that, instead of where the women have come since they’ve had that [experience]. (P34, baseline assessment)

This quote points to the importance for instructors to hold awareness of participants’ trauma experiences while also acknowledging their individual and collective resiliency and accomplishments within the group. Building upon this idea, another participant outlined how the strength of the group lies in their shared understanding and experiences, “It's being able to relate to each other … have a non-judgement environment … just share our stories together and really support each other … through these tough times” (P12, 3-month assessment).

The resilience of the women and the healing power of the collective were evident. A participant described her first few experiences attending the walking group:

The first three times I was like, ugh, this is really hard, ugh, I kind of don’t want to be here. Then everyone starts talking and everyone is kind of having those challenges, so you don’t feel so bad. We all kind of motivate each other and it's nice. (P15, 3-month assessment)

Another participant recognized the struggles women like herself face in being physically active but wanted to recognize the strength gained in showing up as part of the collective. She stated,

People do struggle with it [physical activity], and it is tied into finances and resources I think, but it's also, there is the human ability to overcome all that and it's what we put our mind to we can achieve as long as we stay focused.

## Discussion

To our knowledge, this is the first FPAR approach to cocreating TVIPA programming for women who experience(d) trauma and/or violence. There are a number of physical activity programs, such as trauma-sensitive yoga or sport programs, that have been studied through a trauma-informed lens (Ammann & Matuska, 2014; Bergholz et al., 2016; Clark et al., 2014; Emerson et al., 2009; Smoyer, 2016); these studies have contributed to a foundation of practical knowledge for the effective implementation of TVIPA. Building upon these ideas, our research contributes to the literature and TVIPA community by further examining programs, practices, and policies from the participants’ perspectives and by considering varied physical activities. To create more equitable and accessible resources and physical activity programs for women, participants’ voices and experiences must be central to all aspects of program development, design, and delivery. Understanding, considering, and implementing programs with considerations of sociohistorical and political contexts in which women reside is crucial. Importantly, our use of an intersectional framework allowed us to recognize that inequities are never the result of a single factor, but rather the intersections of multiple social categories and power relations that (re-)interact to shape physical activity access and experiences (Bowleg, 2012). Supported by the themes we identified, we suggest that TVIPA is an effective strategy for engaging pregnant and parenting women who experience(d) trauma. To further examine our four themes and the tenets of TVIPA, we discuss the findings and provide important insights into the application of TVIPA for pregnant and parenting women who experience(d) trauma.

### Trauma Awareness

The importance of the TVIPA tenets to women's participation in programming was made ever clear, beginning with the necessity to first recognize the way that traumatizing circumstances are inherently embedded within social positions, including gender, and access to resources. Physical activity researchers often neglect the impact of interpersonal and structural violence as determinants of physical activity (Giles-Corti & Donovant, 2002). The women in our study detailed the importance of trauma awareness in the delivery of physical activity programming. It is important to preface that offering TVIPA does not require practitioners to provide trauma treatment; nevertheless, they must increase their sensitivity to triggers, attempt to reduce said triggers, and be prepared to watch for and respond to possible trauma reactions. First and foremost, integrating trauma awareness into programming means acknowledging that participants have diverse and complicated histories. Increased recognition of the gendered effects that trauma histories and/or ongoing experiences of violence, particularly GBV, have on individuals can further expand our understanding of trauma in relation to intersecting marginalizing experiences within the physical activity context.

From an intersectional approach, trauma awareness requires programmers to engage in self-reflexivity to recognize and understand how different positionalities and access to resources shape physical activity experiences. Because programming may exclude or exacerbate symptoms related to trauma, it is advised to have adaptations or strategies prepared in advance to address these challenges. Participants identified that some physical activity programs left them feeling unsupported and unsafe; therefore, an interrogation of the ways that traditional approaches to physical activity are connected to, or are complicit in, systems that perpetuate structural violence is necessary. A focus on experiences of violence and subsequent obstacles to participation can help to identify approaches that may best address specific physical and social needs and boundaries to enable accessibility. Finally, this approach requires individuals to recognize the intersections among structural violence, trauma, substance use, and health issues (Monteanti & Thurston, 2015).

### Safety and Trustworthiness

In line with FPAR, the participants referred to power imbalances within mainstream physical activity programs and detailed strategies that service providers can adopt to increase safety and trustworthiness. Safety in this context is not only physical—but it is also cultural and emotional. Disparities based on class, race, ability, and sexuality can impede relationship building; thus, practitioners are encouraged to engage in rigorous self-reflexivity and ensure that they are not perpetuating any discriminatory practices (Browne et al., 2009).

Dominant approaches to physical activity are not appropriate for populations who have experienced trauma or ongoing marginalization (Weissbecker & Clark, 2007). Instead, services should prioritize building relationships and encouraging participants to meet their own goals and celebrate small successes. Adaptations to consider include scheduling additional time before and after programming to allow time for check-ins with participants and opportunities to debrief. Further, creating a consistent or clear schedule for the program is important to provide participants with a snapshot of what to expect. Finally, given the prevalence of GBV among women who live in marginalizing conditions (Covington, 2008), it is paramount to prioritize respect for physical boundaries. Practitioners are strongly encouraged to completely avoid physical touch and instead use no-touch assists as a matter of personal safety and boundaries (Clarke et al., 2014; Emerson, 2015).

Participants also identified additional strategies to increase feelings of safety: introducing new instructors to the program and participants before leading a class; programming in women-only spaces with self-identified female instructors, which has proved useful in other programs for women who have experienced violence (Cole & Ullrich-French, 2017); and ensuring programmers or instructors do not push personal agendas. A fundamental concept of these strategies is that services should, above all, be created to meet the needs of the participants (Partyka, 2019).

### Choice, Collaboration, and Connection

The women in our research overwhelmingly agreed that most existing programs and resources did not meet their needs. Attending to opportunities for choice and collaboration requires recognizing and addressing the barriers that women face to accessing programs, including, but not limited to, cost, childcare, accessible locations, and discrimination. Participants expressed the desire to be included in decision-making processes and program design. Choice and collaboration were also deemed important factors beyond just program development alone, wherein women indicated choice as a priority *during* programming as well.

### Strengths-Based and Capacity Building

When considering the application of strength-based approaches in our TVIPA pilot study, there were three practical ways in which we focused on the community's strengths: first, recognizing accessible and familiar locations for participants; second, drawing on existing physical activity programming and integrating trauma- and violence-informed training and supports to local facilitators, and finally, facilitating the development of new connections among participants. The vast majority of women in the study discussed the importance of establishing healthy relationships with other women who have similar lived experiences in the context of being physically active.

In terms of limitations, the scope of this study was limited to a small geographic location and focused on pregnant and parenting women. Future work should focus on other adult populations and tailor TVIPA to different settings and contexts. The limitation is also a strength of this research, whereby we argue that TVIPA must be context-specific in order to meet the specific needs of the community. Broader lessons learned from this research are certainly applicable to many populations, however, the nuance and unique needs of communities must be addressed.

The data collected for this research was completed prior to the COVID-19 pandemic; since that time, there has been a global upsurge in GBV (Saunders et al., 2021). Further, there is decreased capacity of community organizations, lack of access to support services for women (Khanlou et al., 2020), and decreased physical activity engagement (Christensen et al., 2022). Given the confluence of the aforementioned issues, TVIPA is arguably of greater critical importance to support pregnant and parenting women, than prior to the pandemic.

## Conclusion

Women who experience trauma and/or violence are disproportionately affected by inaccessible physical activity programs and resources. TVIPA is context specific, responsive to community-identified needs, and must address social determinants of physical activity and inequity. An intersectional lens calls for power relations to be central to analysis, which is congruent with FPAR. Through the lens of an intersectional theoretical framework and FPAR, we identified the ways in which TVIPA can mitigate many barriers to participation in physical activity programs. Moreover, a focus on experiences of individual, systemic, and structural violence and subsequent obstacles to participation can help to identify approaches that may enable greater accessibility to physical activity. This study shows that TVIPA is a feasible approach to improve access to physical activity for pregnant and/or parenting women who have experienced trauma and should be considered and employed in diverse community contexts.

## Notes

1. Residential schools in Canada were government-sponsored religious schools that operated for over 160 years with 150,000 Indigenous children. The schools were a system to civilize and assimilate Indigenous people and to “kill the Indian in the child” (Truth and Reconciliation Commission of Canada, 2015, p. 130) by forcibly taking children from their families, communities, and cultures—causing irreparable damage.
